# Bound entanglement is not Lorentz invariant

**DOI:** 10.1038/s41598-023-38217-3

**Published:** 2023-07-11

**Authors:** Paweł Caban, Beatrix C. Hiesmayr

**Affiliations:** 1 Department of Theoretical Physics, University of Lodz, Pomorska 149/153, 90-236 Łódź, Poland; 2 Faculty of Physics, University of Vienna, Boltzmanngasse 5, 1090 Vienna, Austria

**Keywords:** Quantum information, Quantum mechanics

## Abstract

Bound entanglement, in contrast to free entanglement, cannot be distilled into maximally entangled states by two local observers applying measurements and utilizing classical communication. In this paper we ask whether a relativistic observer classifies states according to being separable, bound or free entangled in the same manner as an unboosted observer. Surprisingly, this turns out not to be the case. And that even if the system in a given inertial frame of reference is separable with respect to the partition momenta versus spins. In detail, we show that if the spin state is initially bound entangled, some boosted observers observe their spin states to be either bound entangled, separable or free entangled. This also explains why a general measure of the entanglement property is difficult to find.

## Introduction

Detecting entanglement, even given the full information of the physical state, namely the density matrix is a NP-hard problem^1^ (NP-hard stands for non-polynomial-time hard), because of the existence of bound or PPT (positive partial transposition) entangled states^2^. Those states cannot be detected by taking the partial transpose in one subsystem and finding at least one eigenvalue negative, in which case we are dealing with *free* entanglement. This mathematical property of the density matrix has crucial physical implementation, i.e. an ensemble of free entangled states can always be distilled to maximally entangled states by local operations and classical communication (LOCC), in strong contrast to bound entangled states. Those states can be generated by maximally entangled states, Bell states, but this entanglement is then bounded, i.e. cannot be distilled. This aspect of entanglement gave raise to a lot of speculations why Nature provides us with that kind of entanglement and for what it could be useful^3,4^. Entanglement and other aspects of quantum information theory in the relativistic setting were discussed in many papers, see, e.g.,^5–15^ and references therein. However, up to our best knowledge, the behavior of bound entanglement under Lorentz boosts was not analyzed up to now. One of the reasons is that bound entanglement is difficult to detect. However, recent works^16–18^ have shown some new insights on the structure of bound entangled states in the Hilbert space for the lowest dimensional cases of two qutrits or two ququarts. We use those results in our present work.

**Figure 1:**
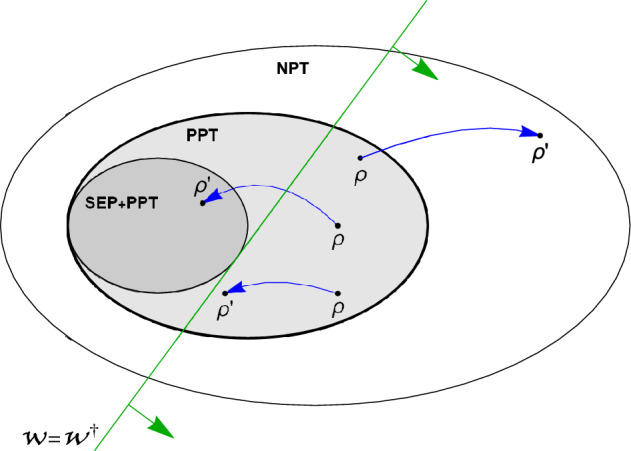
This visualizes the state space and the effect of a relativistic boost on bound entangled state. An entanglement witness $\mathscr {W}$ is visualized as a line separating some entangled states from separable states.

In this paper we analyze how bound entanglement changes under Lorentz boosts. To this end we consider a system of two massive spin-1 particles. In a one inertial frame of reference this system is prepared in a state that is separable with respect to the partition momenta versus spins and the spin part of this state is bound entangled. We show that there exist such states and boosts that the boosted state is also bound entangled or separable or even free entangled. This is also visualized in Fig. 1.

## Methods

### Two-qutrit bound entangled states

Entanglement is a genuine quantum feature of multipartite systems. However, even in a bipartite case complete characterization of entanglement can be given only for systems with dimensions $2\otimes 2$ and $2\otimes 3$. In these dimensions one can fully characterize entanglement with the help of the Peres–Horodecki Positive Partial Transpose (PPT) criterion^19,20^. This criterion says that if $\rho _{AB}^{T_B}$ (where the superscript $T_B$ denotes partial transposition with respect to the system *B*) is not positive semidefinite then the state $\rho _{AB}$ is entangled. For $2\otimes 2$ and $2\otimes 3$ dimensional systems also the inverse is true. However, for higher dimensional systems there exist entangled states $\rho _{AB}$ such that $\rho _{AB}^{T_B}$ is positive semidefinite^2^. Such states are called bound entangled, in opposition to free entangled states for which $\rho _{AB}^{T_B}$ possesses at least one negative eigenvalue. These names—free and bound entanglement—come from the fact that free entanglement can be distilled while it is impossible for bound entanglement.

The detection of bound entanglement is not an easy task—given method can certify entanglement of a certain family of states while it can be useless for other^16^. One of the most useful and easiest in application methods is the realignment or computable cross-norm criterion^21,22^, on which we focus firstly. In the last section we show how those properties of states can be transferred via so called entanglement witnesses, i.e. hermitian observables, to an experimental realization. Let us denote

1 $$\begin{aligned} {{\,{\textsf{Realignment}}\,}}(\rho _{AB})= \log _2 \left( \sum _i \sigma _i(\tilde{\rho }_{AB}) \right) , \end{aligned}$$

where $\sum _i \sigma _i(\tilde{\rho }_{AB})$ is the sum of all the singular values of the realigned matrix $\tilde{\rho }_{AB}$, where $[\tilde{\rho }_{AB}]_{ij,ab}=[\rho _{AB}]_{ia,jb}$. The realignment criterion says that if ${{\,{\textsf{Realignment}}\,}}(\rho _{AB})>0$ then the state $\rho _{AB}$ is entangled. The above statement cannot be inverted—there exist entangled states $\rho$ for which ${{\,{\textsf{Realignment}}\,}}(\rho )<0$. Moreover, this criterion can detect bound entanglement of certain classes of states.

Bound entanglement can be also revealed by other methods like the quasispin criterion^23^ or with the help of entanglement witnesses (here the witnesses constructed with the help of mutually unbiased bases, so called MUB witnesses^24–26^ are useful), which we discuss in the last section.

In this paper we use bound entangled states from the so called magic simplex for which recently a classification into separable, bound entangled and free entangled states was possible with a success probability of $95\%$ for qutrits^17^ and for ququarts of $75\%$^18^. In the $3\otimes 3$ dimensional case this simplex has the form^27,28^

2 $$\begin{aligned} \mathscr {M}_3 \equiv \left\{ \rho = \sum _{k,l=0}^{2} c_{k,l} P_{k,l}~ |~ \sum _{k,l=0}^{2} c_{k,l} = 1, c_{k,l} \ge 0 \right\} , \end{aligned}$$

where $P_{k,l}=|\Omega _{k,l}\rangle \langle \Omega _{k,l}|$ and the Bell states $|\Omega _{k,l}\rangle$ can be generated from $|\Omega _{0,0}\rangle =\frac{1}{\sqrt{3}}(|00\rangle +|11\rangle +|22\rangle )$ via the relation $|\Omega _{k,l}\rangle = W_{k,l}\otimes {\mathbb {I}}_3\; |\Omega _{0,0}\rangle$. In the last equation $W_{k,l}$ are the unitary Weyl operators $W_{k,l} \equiv \sum _{j=0}^{2} \omega ^{j \cdot k}\; |j\rangle \langle j+l|$ with $\omega = e^{\frac{2 \pi i}{3}}$ being the root of unity.

In particular we will use an interesting one parameter state^16^

3 $$\begin{aligned} \rho _b(x) = \sum _{k,l=0}^{2} d_{k,l} |\Omega _{k,l}\rangle \langle \Omega _{k,l}| \end{aligned}$$

with

4 $$\begin{aligned} \begin{pmatrix} d_{0,0}&{}\quad d_{0,1}&{}\quad d_{0,2}\\ d_{1,0}&{}\quad d_{1,1}&{}\quad d_{1,2}\\ d_{2,0}&{}\quad d_{2,1}&{}\quad d_{2,2} \end{pmatrix} = \begin{pmatrix} 2x&{}\quad 0&{}\quad \frac{1}{3}-x\\ 0&{}\quad x&{}\quad \frac{1}{3}-x\\ 0&{}\quad 0&{}\quad \frac{1}{3}-x \end{pmatrix} \end{aligned}$$

and $0\le x \le \tfrac{1}{3}$. This state is PPT for $x\in [0,\frac{2}{15}]$, however, entangled for all values *x* except $x=0$. This means that for $x\in \{0,\frac{2}{15}]$ it is bound entangled, which is detected by the realignment criterion (1) as well as by the later introduced MUB-witness^24–26^.

### Action of Lorentz boosts on quantum states

Bound entanglement can be observed in a two-particle system with at least $3\otimes 3$ dimensions, where in turn the PPT criterion is only necessary but not sufficient for entanglement. Thus, to analyze the behavior of bound entanglement under Lorentz boost, we consider here a system of two massive, relativistic spin-1 particles. We identify the Hilbert space of states of such a particle, $\mathscr {H}$, with the carrier space of the irreducible, unitary massive representation of the Poincaré group for spin 1. The space $\mathscr {H}$ is spanned by the eigenvectors of the four-momentum operator $|k,\sigma \rangle$, where $k=(k^0,{\textbf {k}})$, $k^2={k^0}^2-{\textbf {k}}^2=m^2$, denotes the four-momentum of the particle and $\sigma =-1,0,1$ its spin component along *z*-axis.

We denote here space-time coordinates with Greek indices running from 0 to 3, four-vectors by plain letters, spacial vectors by bold letters, e.g., $k=(k^0,{\textbf {k}})$. The Minkowski tensor is assumed to be $\eta =\text {diag}(1,-1,-1,-1)$. We also use natural units with $c=\hbar =1$.

We use the Lorentz-covariant normalization

5 $$\begin{aligned} \langle k,\sigma |k',\sigma '\rangle = 2k^0\delta ^3\;({\textbf {k}}-{\textbf {k}}^{\prime })\cdot \delta _{\sigma \sigma '}. \end{aligned}$$

The vectors $|k,\sigma \rangle$ can be generated from the standard vector $|\tilde{k},\sigma \rangle$, where $\tilde{k}=m(1,0,0,0)$ is the four-momentum of the particle in its rest frame. We have $|k,\sigma \rangle =U(L_k)|\tilde{k},\sigma \rangle$, where the standard Lorentz boost $L_k$ is defined by relations $k=L_k\tilde{k}$, $L_{\tilde{k}}=\mathbb {I}_4$. The explicit form of the boost $L_k$ reads

6 $$\begin{aligned} L_k = \frac{1}{m} \begin{pmatrix} k^0 &{}\quad {\textbf {k}}^T \\ {\textbf {k}} &{}\quad m\mathbb {I}_3 + \frac{{\textbf {k}}\otimes {\textbf {k}}^T}{m+k^0} \end{pmatrix}. \end{aligned}$$

With the help of the standard Wigner procedure^29–31^ we get

7 $$\begin{aligned} U(\Lambda )|k,\sigma \rangle = \mathscr {D}_{\lambda \sigma }(R(\Lambda ,k))|\Lambda k,\lambda \rangle , \end{aligned}$$

where the Wigner rotation $R(\Lambda ,k)$ is defined as $R(\Lambda ,k)=L_{\Lambda k}^{-1}\Lambda L_k$ and $\mathscr {D}$ is a three dimensional, unitary, irreducible representation of the rotation group. It is well known that in each dimension there exists, up to unitary equivalence, only one unitary, irreducible representation of the rotation group. Therefore, the representation $\mathscr {D}(R)$ is unitary equivalent to *R*

8 $$\begin{aligned} \mathscr {D}(R)=VRV^{\dag },\qquad V^{\dagger} V = \mathbb {I}_3, \end{aligned}$$

and the explicit form of the matrix *V* is the following:

9 $$\begin{aligned} V=\frac{1}{\sqrt{2}} \begin{pmatrix} -1 &{}\quad i &{}\quad 0 \\ 0 &{}\quad 0 &{}\quad \sqrt{2} \\ 1 &{}\quad i &{}\quad 0 \\ \end{pmatrix}. \end{aligned}$$

For more details on spin-1 irreducible, unitary representation of the Poincare group see, e.g., Refs.^29–31^.

For our computations we identify spin projection values $-1,0,1$ with indices of computational basis vectors 0, 1, 2 in the following way: $-1 \leftrightarrow 0$, $0 \leftrightarrow 1$, $1 \leftrightarrow 2$.

## Results

### Bound entanglement under Lorentz boosts

Now, let us consider two inertial frames, $\mathscr {O}$ and $\mathscr{O}^{\prime}$, and let the frame $\mathscr {O}^{\prime}$ move with the velocity ${\textbf {v}}$ with respect to the frame $\mathscr {O}$. In the frame $\mathscr {O}$ we prepare a two-particle state $\rho$. For simplicity we treat momentum degrees of freedom as discrete, i.e. we assume that momenta of the particles are chosen from the finite set $\{k_1,\dots ,k_N\}$. To prove all of our results it is enough to limit to only two momenta $\{k_1,k_2\}$ with

10 $$\begin{aligned} k_{1,2}=(k^0,\pm |{\textbf {k}}|,0,0) \end{aligned}$$

Consequently, our total state under interest acts in $(2\otimes 2)_{\textsf{mom}}\otimes (3\otimes 3)_{\textsf{spin}}=36$ dimensional space, i.e. in ${\mathbb {C}}^{36}$. Of course, also higher dimensions in the momentum space are possible, but not necessary to obtain our results. Moreover, without loss of generality we can take

11 $$\begin{aligned} m=1,\quad k^0 = 1+E,\quad |{\textbf {k}}|=\sqrt{E(2+E)}, \end{aligned}$$

where *E* is a kinetic energy of the particle. Thus, the most general two-particle state we consider is of the form

12 $$\begin{aligned} \rho = \sum \rho _{mn,m^{\prime} n^{\prime} }^{\sigma \lambda ,\sigma ^{\prime} \lambda ^{\prime} } |k_m,k_n;\sigma ,\lambda \rangle \langle k_{m^{\prime} },k_{n^{\prime} };\sigma ^{\prime} ,\lambda ^{\prime} |, \end{aligned}$$

where $m,n,m^{\prime} ,n^{\prime} =1,2$, $\sigma ,\lambda ,\sigma ^{\prime} ,\lambda ^{\prime} =0,1,2$ and $\rho _{mn,m^{\prime} n^{\prime} }^{\sigma \lambda ,\sigma ^{\prime} \lambda ^{\prime} }$ fulfill all necessary conditions to guarantee that $\rho$ is a valid density matrix. We have also reordered the products of momentum and spin components, i.e. $|k,p;\sigma ,\lambda \rangle =|k,p\rangle \otimes |\sigma ,\lambda \rangle \equiv |k,\sigma \rangle \otimes |p,\lambda \rangle$.

The state $\rho$ as seen from the frame ${\mathscr{O}}^{\prime}$ has the following form

13 $$\begin{aligned} \rho ^{\prime} = [U(\Lambda ({\textbf {e}},\xi ))\otimes U(\Lambda ({\textbf {e}},\xi ))] \rho [U(\Lambda ({\textbf {e}},\xi ))\otimes U(\Lambda ({\textbf {e}},\xi ))]^{\dagger} . \end{aligned}$$

Here $\Lambda ({\textbf {e}},\xi )$ is the Lorentz boost in the direction ${\textbf {e}}={\textbf {v}}/|{\textbf {v}}|$ with rapidity $\xi$, $\tanh \xi =-|{\textbf {v}}|$, joining frames $\mathscr {O}$ and $\mathscr{O}^{\prime}$. Its explicit form is the following:

14 $$\begin{aligned} \Lambda ({\textbf {e}},\xi ) = \begin{pmatrix} \cosh \xi &{}\quad {\textbf {e}}^T \sinh \xi \\ {\textbf {e}} \sinh \xi &{}\quad \mathbb {I}_3 + (\cosh \xi - 1){\textbf {e}}\otimes {\textbf {e}}^T \end{pmatrix}. \end{aligned}$$

The action of $U(\Lambda )$ on basis states of the space ${\mathscr {H}}$ is given in Eq. (7).

The spin parts of the states $\rho$ and $\rho ^{\prime }$ (i.e. $\rho _\textsf{spin}$ and $\rho _{\textsf{spin}}^{\prime }$, respectively) we obtain by tracing out momentum degrees of freedom and normalizing the result since in covariant normalization (5) basis vectors are orthogonal but not orthonormal. Of course in the frame ${\mathscr{O}}^{\prime}$ momenta of the particles belong to the set $\{k_{1}^{\prime} ,k_{2}^{\prime} \}$, where $k_{i}^{\prime} = \Lambda ({\textbf {e}},\xi ) k_i$, $i=1,2$.

#### Pure momentum part of the state

As the first case we consider the simple situation when in the frame $\mathscr {O}$ we prepare a two-particle state

15 $$\begin{aligned} \rho =\sum _i p_i |\psi _i\rangle \langle \psi _i|,\quad p_i\ge 0\quad \textrm{with}\quad \sum _i p_i=1, \end{aligned}$$

where

16 $$\begin{aligned} |\psi _i\rangle = |\psi ^{\textsf{mom}}\rangle \otimes |\varphi _i^\textsf{spin}\rangle , \quad |\psi _i\rangle \in \mathscr {H}^\textsf{mom}\otimes \mathscr {H}^\textsf{spin}, \end{aligned}$$

and $|\psi ^{\textsf{mom}}\rangle$, $|\varphi _i^\textsf{spin}\rangle$ are momentum and spin parts of the state $|\psi _i\rangle$, respectively. Thus, we assume that the momentum parts of all of the states $|\psi _i\rangle$ are identical. Notice that the full state $\rho$ defined in Eqs. (15, 16) is separable with respect to the partition: momenta versus spins (although it is not the most general separable state, the general separable state we consider in the next section). Therefore, we can write (15) as

17 $$\begin{aligned} \rho = |\psi ^{\textsf{mom}}\rangle \langle \psi ^{\textsf{mom}}| \otimes \left( \sum _i p_i |\varphi _i^\textsf{spin}\rangle \langle \varphi _i^\textsf{spin}| \right) \equiv |\psi ^{\textsf{mom}}\rangle \langle \psi ^{\textsf{mom}}| \otimes \rho _{\textsf{spin}}, \end{aligned}$$

and of course $\rho _{\textsf{spin}} = {{\,\textrm{Tr}\,}}^{\textsf{mom}}(\rho )$. We are interested in the situation when $\rho _{\textsf{spin}}$ ia a bound entangled state.

The most general form of $|\psi ^{\textsf{mom}}\rangle$ in our case reads

18 $$\begin{aligned} |\psi ^{\textsf{mom}}\rangle = \sum _{i,j=1}^{2} a_{ij} |k_i,k_j\rangle , \quad \sum _{i,j=1}^{2} |a_{ij}|^2=1. \end{aligned}$$

Now, we boost the state (17) and the spin part of the boosted state has the form

19 $$\begin{aligned} \rho _{\textsf{spin}}^{\prime} = {{\,\textrm{Tr}\,}}^{\textsf{mom}}(\rho ^{\prime} ) = \sum _{i,j=1}^{2} |a_{ij}| \left[ \mathscr {D}^T(\Lambda ,k_i) \otimes \mathscr {D}^T(\Lambda ,k_j) \right] \rho _{\textsf{spin}} \left[ \mathscr {D}^*(\Lambda ,k_i) \otimes \mathscr {D}^*(\Lambda ,k_j) \right] . \end{aligned}$$

Following^2^ and using the property

20 $$\begin{aligned} \left[ (A\otimes B)\rho (C\otimes D) \right] ^{T_B} = (A\otimes D^T) \rho ^{T_B} (C\otimes B^T) \end{aligned}$$

we easily see that if $\rho _{\textsf{spin}}$ is a PPT state then also $\rho _{\textsf{spin}}^{\prime}$ is also PPT. Thus, if the spin state of the two-particle state (17) is bound entangled then for all other inertial observers this state is PPT, i.e. also bound entangled or separable. We now show that both of these cases can be realized. To this end we assume that

21 $$\begin{aligned} |\psi ^{\textsf{mom}}\rangle = |\psi ^{\textsf{mom}}_1\rangle = \frac{1}{\sqrt{2}} (|k_1,k_2\rangle + |k_2,k_1\rangle ). \end{aligned}$$

where $k_1,k_2$ are given in Eq. (10). Moreover, as a spin part of the state (17) we take the state $\rho _b$ defined in Eq. (3). Thus, the state chosen in the frame $\mathscr {O}$ has the form

22 $$\begin{aligned} \rho _1(x) = |\psi ^{\textsf{mom}}_1\rangle \langle \psi ^{\textsf{mom}}_1| \otimes \rho _b(x). \end{aligned}$$

We further choose the boost direction ${\textbf {e}}=(0,0,1)$ (compare with Eq. (14)) and kinetic energy of a particle $E=1$ (compare with Eq. (11)).

**Figure 2:**
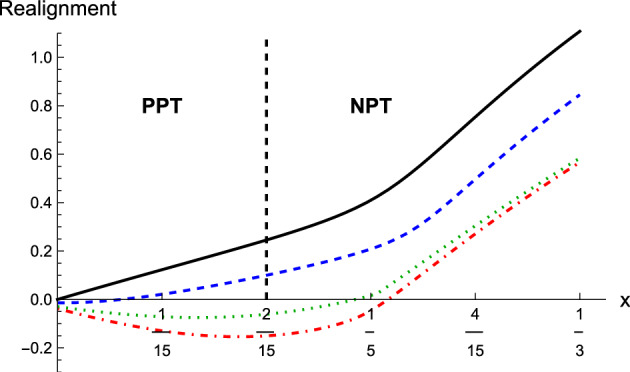
Value of the base-2 log of the sum of all singular values of the realigned spin density matrix for unboosted state (black, solid line) and states boosted with rapidity $\xi =0.5$ (blue, dashed line), $\xi =0.8$ (green, dotted line), $\xi =1$ (red, dashed-dotted line).

In Fig. 2 we plotted the value of the base-2 log of the sum of all singular values of the realigned spin density matrix versus *x* for the unboosted and boosted state (22). According to the realignment criterion^21^, if this value is positive the state is entangled. We can see that for the unboosted state the realignment criterion detects entanglement for all values of *x* except 0, in contrast to the boosted state $\rho _{\textsf{spin}}^{\prime} (x,\xi )$ where it detects entanglement only for $x\in (x_0,1/3]$. Thus, in the considered case there always exist such a *x* and $\xi$ that the bound entangled state $\rho _{\textsf{spin}}=\rho _b(x)$ after boost is also bound entangled.

**Figure 3:**
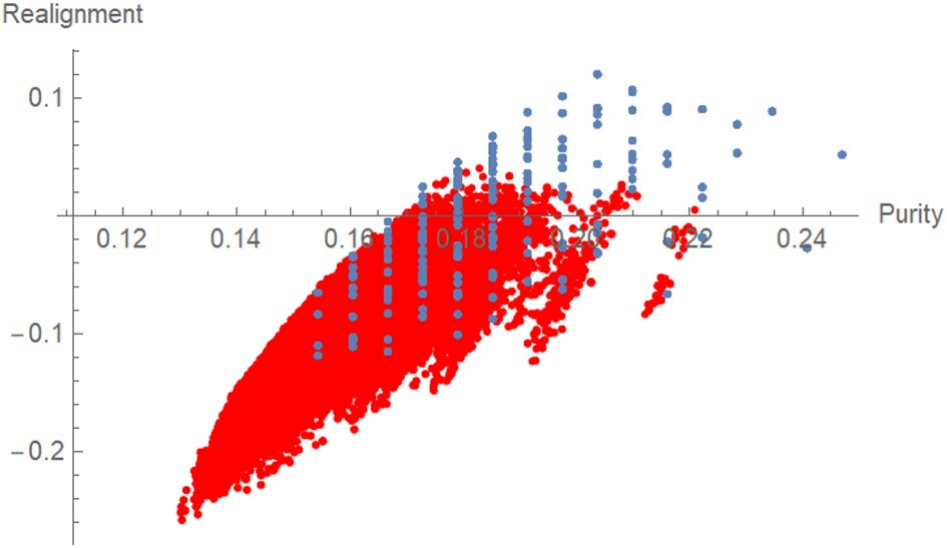
This picture visualizes the purity ${{\,\textrm{Tr}\,}}\rho ^2$ versus the realignment $\sum _i \sigma _i(\tilde{\rho }_{AB})-1$ of 156600 bound entangled magic simplex states^16^. Those states result in 2228 (blue) points and if boosted with $\xi =0.8$ they result in 87116 (red) points for our particular choice of boost. This shows that the relativistic boost changes the amount of entanglement (in this case measured by the realignment criterion) not in dependence of the initial amount of entanglement. This is also the case if other entanglement measures or purity measures are chosen.

In Fig. 3 we plotted the purity ${{\,\textrm{Tr}\,}}(\rho ^2)$ versus realignment of 156600 magic bound entangled states on a grid in the magic simplex^16^, where only some are detected by the realignment criterion. The other ones are detected by different criteria. After the boost with $\xi =0.8$ the purity decreases and fewer states are still detected by the realignment criterion. Thus the interesting question is: Does a boost of a bound entangled state result sometimes also in separable states?

To find that out we can try to apply other entanglement criteria such as the quasispin criterion^23^ or the MUB witness^24^ which failed. Thus, for e.g. $x=\frac{1}{15},\xi =0.8$ we tried to show that $\rho _{\textsf{spin}}^{\prime}$ is separable and were successful. We performed this by numerically minimizing the Hilbert-Schmidt distance $\big [{{\,\textrm{Tr}\,}}\big (\big (\sum _{i=1}^{k} p_i^\textsf{sep} |\psi ^\textsf{sep}_i\rangle \langle \psi ^\textsf{sep}_i| -\rho _{\textsf{spin}}^{\prime} \big (\tfrac{1}{15},\tfrac{4}{5}\big )\big )^2\big )\big ]^{1/2}$, where $p_i^\textsf{sep}>0$, $\sum _{i=1}^{k} p_i^\textsf{sep}=1$, and $|\psi ^\textsf{sep}_i\rangle$ represent separable states, for some large enough *k*. For $k=10$ we found probabilities $p_i^\textsf{sep}$ and vectors $|\psi ^\textsf{sep}_i\rangle$ such that

23 $$\begin{aligned} \left[ {{\,\textrm{Tr}\,}}\left( \left( \sum _{i=1}^{10} p_i^\textsf{sep} |\psi ^\textsf{sep}_i\rangle \langle \psi ^\textsf{sep}_i| -\rho _{\textsf{spin}}^{\prime} \left( \tfrac{1}{15},\tfrac{4}{5}\right) \right) ^2\right) \right] ^{1/2} \approx 7\times 10^{-8}, \end{aligned}$$

i.e. practically equal to zero. The explicit form of probabilities $p_i^\textsf{sep}$ and vectors $|\psi ^\textsf{sep}_i\rangle$ we give in Supplementary Material. We were also successful for other $\xi$ values and other *x* values like $x=\frac{1}{10}$, but not for $x>\frac{2}{15}$, the NPT area.

In summary, we have found at least some states which are after the boost separable, which is apparently against the intuition if it depends on the boost of the observer whether a state is classified as bound entangled or separable.

#### General separable state momenta versus spins

Now, let us assume that in the frame $\mathscr {O}$ we prepare the most general state that is separable in the partition momenta versus spins, i.e.

24 $$\begin{aligned} \rho = \sum _i p_i\; \rho _{\textsf{mom}}^i \otimes \rho _{\textsf{spin}}^i, \end{aligned}$$

where $p_i>0$, $\sum _i p_i=1$. The spin part of this state has the form

25 $$\begin{aligned} \rho _{\textsf{spin}} = {{\,\textrm{Tr}\,}}_{\textsf{mom}}(\rho ) = \sum _i p_i\; \rho _{\textsf{spin}}^i. \end{aligned}$$

As previously, we are interested in the situation when the whole state $\rho _{\textsf{spin}}$ is bound entangled.

Now, we boost the state (24) and calculate the spin part of the boosted state as $\rho _{\textsf{spin}}^{\prime} = {{\,\textrm{Tr}\,}}^{\textsf{mom}}(\rho ^{\prime} )$ and we obtain

26 $$\begin{aligned} \rho _{\textsf{spin}}^{\prime} = \sum _{i,m,n} p_i c_{mn,mn}^i\; \left[ \mathscr {D}^T(\Lambda ,k_m) \otimes \mathscr {D}^T(\Lambda ,k_n) \right] \rho _{\textsf{spin}}^i \left[ \mathscr {D}^*(\Lambda ,k_m) \otimes \mathscr {D}^*(\Lambda ,k_n) \right] , \end{aligned}$$

where

27 $$\begin{aligned} \rho _{\textsf{mom}}^i = \sum _{m,n,m^{\prime} ,n^{\prime} } c_{mn,m^{\prime} n^{\prime} }^i\; |k_m,k_n\rangle \langle k_{m^{\prime} },k_{n^{\prime} }|, \end{aligned}$$

i.e. $$c_{mn,mn}^i>0$$, $$\sum _{m,n} c_{mn,mn}^i =1$$.

Now, we ask whether the transformation (26), the relativistic boost, preserves PPT. However, applying Eq. (20) we have to take into account that the whole state $\sum _i p_i \rho _{\textsf{spin}}^i$ is PPT but some of the states $\rho _{\textsf{spin}}^i$ can be NPT. Next, factors $c_{mn,mn}^i$ change relative weights in the sum (26). Thus, we cannot conclude that (26) preserves PPT. In fact, we can find such states and boosts that (25) is bound entangled while (26) is free entangled. For example, let us consider the following state:

28 $$\begin{aligned} \rho _0(p,x) = p\; \rho _1(x) + (1-p)\; \rho _2, \end{aligned}$$

where $0\le p \le 1$, $\rho _1(x)$ is given in Eq. (22) and

29 $$\begin{aligned} \rho _2 = |k_1,k_2\rangle \langle k_1,k_2| \otimes |\varphi _\textsf{spin}\rangle \langle \varphi _\textsf{spin}|, \end{aligned}$$

with

30 $$\begin{aligned} |\varphi _\textsf{spin}\rangle = \sum _{k,l=0}^{2} a_{k,l} |\Omega _{k,l}\rangle , \end{aligned}$$

and

31 $$\begin{aligned} \begin{pmatrix} a_{0,0}&{}\quad a_{0,1}&{}\quad a_{0,2}\\ a_{1,0}&{}\quad a_{1,1}&{}\quad a_{1,2}\\ a_{2,0}&{}\quad a_{2,1}&{}\quad a_{2,2} \end{pmatrix} = \begin{pmatrix} 0&{}\quad 2/9&{}\quad 2/9\\ 0&{}\quad 2/9&{}\quad 1/18\\ 5/18&{}\quad 0&{}\quad 0 \end{pmatrix}. \end{aligned}$$

The spin part of the state $\rho _0(0.04,\tfrac{7}{60})$, $\rho _0^{\textsf{spin}}(0.04,\tfrac{7}{60})$ is PPT and

32 $$\begin{aligned} {{\,{\textsf{Realignment}}\,}}\left( \rho _0^{\textsf{spin}} \left( 0.04,\tfrac{7}{60}\right) \right) = 0.183 >0, \end{aligned}$$

thus it is bound entangled.

Now, if we boost this state in the direction ${\textbf {e}}=(0,0,1)$ with $\xi =0.95$ we obtain the state $\rho _0^{\prime} (0.04,\tfrac{7}{60})$. The spin part $\rho _0^{\prime ,\textsf{spin}}(0.04,\tfrac{7}{60})$ is not PPT, thus it is free entangled and obviously no longer bound entangled.

In summary, we have shown that starting from a bound entangled state, we can boost to a separable, bound entangled or an free entangled state. In the next section we discuss how those counter-intuitive classifications of inertial observers relates to the principle that the physics observed, i.e. probabilities and expectation values, should be Lorentz invariant.

## Discussion

Obviously, the physics for every observer should be the same. In our case this means that the values that a boosted or not boosted observer obtains by computing ${{\,\textrm{Tr}\,}}(\mathscr {O}\rho )$ for some observable $\mathscr {O}=\mathscr {O}^{\dagger}$ are identical. An observable $\mathscr {W}$ for which

33 $$\begin{aligned} \min _{\rho _{\textsf{sep}}}({{\,\textrm{Tr}\,}}(\mathscr {W}\rho _{\textsf{sep}})) \le {{\,\textrm{Tr}\,}}(\mathscr {W}\rho ) \le \max _{\rho _{\textsf{sep}}}({{\,\textrm{Tr}\,}}(\mathscr {W}\rho _{\textsf{sep}})) \end{aligned}$$

does not hold for all states $\rho$ is called an entanglement witness. The upper and lower bounds define the so called separability window of the witness and can be obtained by mirroring the witness^32^. A particular witness, decomposable into mutually unbiased bases (MUBs), is capable to detect bound entanglement^24–26^ and as it contains a recipe how to realize it experimentally, it gave raise to the first experiment detecting bound entanglement in bipartite systems^33^. We will use this witness to show how we can solve the apparent puzzle. Obviously, we have

34 $$\begin{aligned} {{\,\textrm{Tr}\,}}(\mathscr {W}\rho )= {{\,\textrm{Tr}\,}}(\mathscr {W}^{\textsf{boosted}}\rho ^{\textsf{boosted}}) \end{aligned}$$

for any chosen boost on the *total* space. But this does not mean that this is the case also for the subsystem, i.e. an partition into first and second momentum versus first and second spin or first momentum and first spin versus second momentum second momentum nor first momentum and second spin versus second momentum and first spin nor in particle A (first momentum/first spin) versus particle B (second momentum/first spin) as we show in the following (for more details on the behavior of entanglement under different partitions see, e.g., Ref.^10^).

The MUB-witness for two qudits that is capable of detecting bound entanglement by the upper bound is defined by

35 $$\begin{aligned} {{\,\textrm{Tr}\,}}(\mathscr {W}\rho ) = \sum _{i=0}^{d-1} \langle i_1|\otimes \langle (i_1+1)^*| \rho |i_1\rangle \otimes |(i_1+1)^*\rangle +\sum _{k=2}^{d+1}\sum _{i=0}^{d-1} \langle i_k| \otimes \langle i_k^*| \rho |i_k\rangle \otimes |i_k^*\rangle , \end{aligned}$$

for which the orthonormal basis $\{i_k\}_{k=1}^{d+1}$ are mutually unbiased $|\langle i_k|j_l\rangle |^2=\delta _{k,l} \delta _{i,j}+(1-\delta _{k,l})\frac{1}{d}$. Thus we can boost each MUB vector and by that achieve the observable $\mathscr {W}^{\textsf{boosted}}$ that is seen by a boosted observer. On the total space we find for our particular example (optimization of the lower and upper bound via the composite parameterization^34^)

36 $$\begin{aligned} -\frac{3}{4}\le {{\,\textrm{Tr}\,}}(\mathscr {W}\rho ) = {{\,\textrm{Tr}\,}}(\mathscr {W}^{\textsf{boosted}}\rho ^{\textsf{boosted}}) =\frac{1}{2}+\frac{x}{4}\le \frac{3}{4}\nonumber \\ \end{aligned}$$

for every boost value $\xi$. This inequality is obviously not violated since our total state was chosen to be separable (momentum versus spin).

Obviously, the situation changes when we consider the spin or momentum subspaces, which we achieve by partial tracing over the respective subsystems. For the spin part we find for the unboosted case

37 $$\begin{aligned} \frac{2}{3}\;\le \; {{\,\textrm{Tr}\,}}({{\,\textrm{Tr}\,}}_{\textsf{mom}} W {{\,\textrm{Tr}\,}}_{\textsf{mom}} \rho )=2+x\;\le 2 \end{aligned}$$

which is obviously violated if $x\not =0$. And e.g. for $\xi =0.8$ we have

38 $$\begin{aligned} 0.763 \;\le \; {{\,\textrm{Tr}\,}}({{\,\textrm{Tr}\,}}_{\textsf{mom}} W^{\textsf{boosted}} {{\,\textrm{Tr}\,}}_{\textsf{mom}} \rho ^{\textsf{boosted}}) \;=\; 1.694 + 0.641 x\;\le \;1.985, \end{aligned}$$

i.e. no violation for any *x*. Consequently, if one ignores in the boosted case the momentum degrees of freedom, one does not consider the full experimental situation and this explains why an unboosted bound state becomes a separable, bound entangled or free entangled one when ignoring part of the systems, though, against our intuition, the momentum part of the witness is just the unity operator for any boost.

In this paper we analyzed the behavior of the bound entangled states under Lorentz boosts. To this end we considered a system of two spin-1, massive particles. In a given inertial frame of reference this system is prepared in a state which is separable with respect to the partition momenta/spins and such that its spin part is bound entangled. Next, we boost this state to a different inertial frame of reference and analyze the entanglement of its spin part. We showed that the boosted state can be also bound entangled. However, we were also able to find such states and boosts that the boosted state is separable or even free entangled. Thus, surprisingly, we found that Lorentz boosts can activate bound entanglement.

In the last section we explained how such a counter-intuitive classification of different inertial observers relates to the principle that measurement outcomes are invariant under Lorentz boosts. Boosting a state means that one boosts also the observable which means taking the trace gives the same result. But ignoring the momentum part, the situation changes drastically and leads to all three separability possibilities as we have shown by exemplary boosts and states.

From the purely practical point of view, we have presented a method to systematically produce different bound entangled states by application of different relativistic boosts, which may further be explored to reveal the very Nature of bound entanglement.

## Supplementary Information

Supplementary Information.

## Data Availability

Numerical data necessary to produce figures were taken from published works. The data are available from the corresponding author on reasonable request. No other data were created during the preparation of the paper.
