# Supplementary material for: Bound entanglement is not Lorentz invariant
Source: Sci Rep. 2023 Jul 11;13:11189. doi: 10.1038/s41598-023-38217-3 (PMC10336123; doi:10.1038/s41598-023-38217-3)
Supplement: Supplementary file 1 — Supplementary Information. [file 41598_2023_38217_MOESM1_ESM.pdf]

Supplementary material for "Is bound entanglement Lorentz invariant?"

Paweł Caban and Beatrix C. Hiesmayr

Below we give an explicit form of probabilities p_i^{sep} and states $|\psi_i^{\mathsf{sep}}\rangle$ for which

$$\left[\mathrm{Tr}\left(\left(\sum_{i=1}^{10} p_i^{\mathsf{sep}}|\psi_i^{\mathsf{sep}}\rangle\langle\psi_i^{\mathsf{sep}}| - \rho'_{\mathsf{spin}}\left(\tfrac{1}{15},\tfrac{4}{5}\right)\right)^2\right)\right]^{1/2} \approx 7\times 10^{-8}, \tag{1}$$

where $\rho'_{\mathsf{spin}}(\frac{1}{15},\frac{4}{5})$ is a spin part of a boosted state (22) for $x=\frac{1}{15}$, $\xi=\frac{4}{5}$.

$$\{p_1^{\mathsf{sep}}, p_2^{\mathsf{sep}}, \ldots, p_{10}^{\mathsf{sep}}\} = \{0.1430992, 0.0734831, 0.0852185, 0.1018063, 0.1491225, 0.0693471, 0.0776687, 0.1398679, 0.0991522, 0.0612343\} \tag{2}$$

$$\psi_1^{\mathsf{sep}} = \begin{pmatrix} -0.0430823 \\ 0.0167110+0.0537862i \\ -0.8497352-0.5175507i \\ -0.0005030-0.0027742i \\ -0.0032683+0.0017040i \\ 0.0234053-0.0607592i \\ 0.0012085-0.0001888i \\ -0.0007044-0.0014356i \\ 0.0261032+0.0107947i \end{pmatrix}, \psi_2^{\mathsf{sep}} = \begin{pmatrix} -0.1680125 \\ -0.0326983-0.4851121i \\ 0.1438131-0.5714999i \\ -0.0775247+0.0557386i \\ -0.1760249-0.2129939i \\ -0.1232379-0.3114132i \\ -0.0481015+0.0804443i \\ -0.2416331-0.1232304i \\ -0.2324608-0.2324767i \end{pmatrix}, \psi_3^{\mathsf{sep}} = \begin{pmatrix} 0.2547478 \\ 0.2422784-0.0536082i \\ -0.1178008+0.0076515i \\ 0.4163048+0.0276915i \\ 0.4017548-0.0612697i \\ -0.1933399-0.0003012i \\ -0.4580805-0.1157550i \\ -0.4600175-0.0136921i \\ 0.2153030+0.0397689i \end{pmatrix} \tag{3}$$

$$\psi_4^{\mathsf{sep}} = \begin{pmatrix} 0.2077491 \\ -0.2713063+0.3203387i \\ 0.5379288-0.2613437i \\ -0.1363512-0.0707598i \\ 0.2871735-0.1178391i \\ -0.4420709-0.0116930i \\ 0.0892001-0.0088623i \\ -0.1028240+0.1491157i \\ 0.2198189-0.1351591i \end{pmatrix}, \psi_5^{\mathsf{sep}} = \begin{pmatrix} -0.0015317 \\ 0.0236887+0.0010515i \\ 0.0009902-0.0043033i \\ 0.0021656-0.0073004i \\ -0.0385048+0.1114204i \\ 0.0191106+0.0108040i \\ 0.0626658-0.0053981i \\ -0.9728846+0.0404651i \\ -0.0253473+0.1795503i \end{pmatrix}, \psi_6^{\mathsf{sep}} = \begin{pmatrix} -0.3732019 \\ -0.2337885+0.1894137i \\ -0.3174714+0.3648181i \\ -0.2753247-0.1903291i \\ -0.2690733+0.0205078i \\ -0.4202637+0.1072325i \\ -0.0215085-0.2204501i \\ -0.1253603-0.1271824i \\ -0.2337944-0.1665049i \end{pmatrix}, \tag{4}$$

$$\psi_7^{\mathsf{sep}} = \begin{pmatrix} 0.0437898 \\ -0.0790503+0.1006166i \\ -0.0675530-0.0621037i \\ -0.1008128+0.0029161i \\ 0.1752890-0.2369033i \\ 0.1596559+0.1384763i \\ -0.0287347+0.2426421i \\ -0.5056504-0.5040468i \\ 0.3884482-0.3335631i \end{pmatrix}, \psi_8^{\mathsf{sep}} = \begin{pmatrix} -0.2327074 \\ -0.0014722-0.0295408i \\ 0.0033542-0.0039737i \\ -0.3230604+0.9062410i \\ -0.1170858-0.0352773i \\ -0.0108184-0.0185789i \\ -0.0454358+0.0419600i \\ -0.0056140-0.0055023i \\ -0.0000616-0.0013807i \end{pmatrix}, \psi_9^{\mathsf{sep}} = \begin{pmatrix} 0.4046742 \\ -0.1996496-0.1249451i \\ -0.1091446+0.0005520i \\ -0.5066867+0.3689981i \\ 0.3639082-0.0256066i \\ 0.1361550-0.1002136i \\ -0.3127123+0.2324570i \\ 0.2260516-0.0181333i \\ 0.0840245-0.0631225i \end{pmatrix}, \tag{5}$$

$$\psi_{10}^{\mathsf{sep}} = \begin{pmatrix} -0.3632900 \\ -0.0910701+0.0798075i \\ -0.0632746-0.1351573i \\ 0.1483556-0.5829032i \\ -0.0908620-0.1787139i \\ 0.2427008-0.0463310i \\ -0.2349668+0.4820461i \\ 0.0469939+0.1724576i \\ -0.2202634-0.0034579i \end{pmatrix}. \tag{6}$$
